# The Prevalence of Gastrointestinal Parasites and Seroprevalence of *Toxoplasma gondii* in Captive Ocelots (*Leopardus pardalis)* in Trinidad, West Indies

**DOI:** 10.1155/2021/8820548

**Published:** 2021-05-26

**Authors:** Alissa Bally, Stacy Francis-Charles, Tariq Ackbar, Yadel Beharrylal, Roxanne Charles, Asoke Basu, Rod Suepaul

**Affiliations:** ^1^Department of Basic Sciences, School of Veterinary Medicine, University of the West Indies, St. Augustine, Trinidad and Tobago; ^2^Small Animal Clinic, School of Veterinary Medicine, St. Georges University, True Blue, Grenada; ^3^Veterinarian in Private Practice, Caroni, Trinidad and Tobago

## Abstract

This study was conducted from November 2010 to June 2011 to determine the prevalence of gastrointestinal parasites and the seroprevalence of *Toxoplasma gondii* in captive ocelots (*Leopardus pardalis*) in Trinidad. Faecal samples were collected and analyzed using faecal flotation to identify helminth ova and protozoan cysts and oocysts. Serum samples from captive ocelots were screened for *T. gondii* using a latex agglutination test kit. Of the 19 ocelots examined, the most prevalent parasites noted were ova of ascarids, strongyles, and *Capillaria* spp. The serum of three of the 13 (23.1%) ocelots tested was positive for *T. gondii* antibodies. These ocelots are therefore a potential source of *T. gondii* infection to both humans and other animals. This is the first documented report of endoparasites in local captive ocelots within Trinidad and provides useful data to support further research of the captive and wild populations.

## 1. Introduction

The ocelot, *Leopardus pardalis*, is the largest felid of the genus *Leopardus*. The pelage is short and thick with a variable coloration that depends on the habitat in which it is found. Distinctive facial patterns allow relatively easy recognition of individuals [1]. Ocelots commonly inhabit the tropical rainforests of Trinidad and Central and South America. They can also be found in marshes, mangroves, thorn scrub regions, and savannah grasslands. They spend most of their time in elevated regions below 1200 meters [1–3]. The diet of ocelots consists mainly of small rodents with some medium-sized animals, reptiles, birds, and aquatic species including crustaceans [4, 5]. These felids are thus very important in neotropical ecosystems, since they are apex predators in the regulation of prey populations [4]. Ocelots are generally not a threat to man since they prefer to hide from or evade human encounters naturally. In Trinidad, ocelots have been reported to be sighted in both northern and southern regions and are notorious for the predation of domestic fowls. These felids are designated as an environmentally sensitive species providing legislative protection for the species. A few that were trapped by hunters in the past have been relocated to the state zoo and other wildlife protection facilities.

Parasitic infections in captive wildlife can result in death to the affected animals, act as a predisposing factor for the development of secondary infectious diseases and exert a negative impact on reproduction. This is especially important in endangered species [6]. *Leopardus pardalis* is the host to a diversity of endoparasites including *Taenia* spp., strongyles, *Paragonimus* spp., *Toxocara cati*, *Capillaria* spp., spirurids, *Aelurostrongylus abstrusus*, acanthocephalans (e.g., *Oncicola* spp.), *Hammondia pardalis*, and *Isospora* spp. [1]. They are also known to host *Toxoplasma gondii*, *Ancylostoma tubaeforme*, *Uncinaria* spp., *Crenosoma* spp., and *Spirometra* spp. [7].

*Toxoplasma gondii* is a zoonotic protozoan parasite that is found in a wide range of mammals and birds. Toxoplasmosis is responsible for abortions in livestock especially sheep and is a common cause of pathology in marsupials [8]. Toxoplasmosis in humans causes mental retardation, seizures, blindness, and death when transmitted congenitally and may be fatal to immunocompromised individuals [9, 10]. Felids are the sole definitive hosts in which the parasite can complete both sexual and asexual stages. Previous reports from neighboring Latin American countries have documented *Toxoplasma gondii* in ocelots [7, 11]. Oocysts shed in ocelot faeces can become infective to other mammalian hosts (including cats, dogs, and humans) via the faecal-oral route [7, 11–13]. Ocelots prey on small mammals, including agouti and rodents, which can act as intermediate hosts with bloodborne tachyzoites and tissue bradyzoite cysts which are infective to felids and other intermediate hosts through predation and carnivorism [14].

This current study therefore aims to identify the endoparasite species which may be found within the captive ocelot population in Trinidad and determine the serological status of *Toxoplasma gondii* in these felids. The ultimate goal is to create and increase the awareness to the public and scientific community of the potential parasitic infections of veterinary and public health significance that can be transmitted by ocelots.

## 2. Materials and Methods

**Figure 1:**
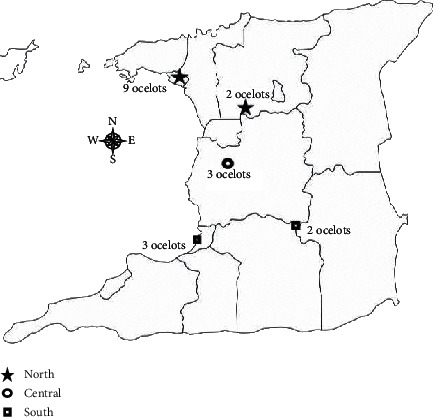
Map of Trinidad displaying the geographic locations of captive ocelots sampled throughout this study.

Ethical approval was granted by the University of the West Indies Campus Research Ethics Committee prior to commencement of this study. A listing of persons holding legal licenses for captive ocelots was sourced from the Forestry Division, Ministry of Agriculture, Land, and Forestry of Trinidad and Tobago. This list revealed five legal captive locations within North, Central, and South Trinidad from which all owners were contacted. The objectives of the study and samples needed were then explained to willing participants. Site visits to these locations showcased ocelots in apparent good health. The cats were kept in cages and maintained in clean, sanitary conditions. Based on locality, 11 ocelots were obtained from North Trinidad from two sites, three ocelots were obtained from Central Trinidad from one site, and five were obtained from two sites on the southern end of the island (Figure 1).

Faecal samples were collected from 17 wild-caught and two captive-born ocelots at all participating sites. Blood samples were taken from only 13 cats dispersed over three of the five sites based on the owner's consent.

### 2.1. Fecal Specimen Collection

Faecal samples were collected from 19 individual animal enclosures and placed in clean plastic bags or plastic faecal cups. To minimize handling stress of the cats, freshly voided faecal samples no older than 12 hours were collected off the ground of enclosures. The samples were then transported in a cooler with ice packs to the Parasitology Laboratory at the University of the West Indies-School of Veterinary Medicine (UWI-SVM) for parasitological analysis. Samples were refrigerated at 4°C for further processing for a maximum of 72 hours.

### 2.2. Flotation Technique

Three grams of faeces were weighed and mixed with 45 ml of 33.3% zinc sulphate solution and then filtered into a clean faecal cup using a tea strainer and gauze. The supernatant was added to a floatation vial until a positive meniscus was formed. A coverslip was placed on the meniscus and left undisturbed for 10–15 minutes. The coverslip was then removed and placed onto a microscopic slide. Each slide was examined microscopically at 10x and 40x objectives for detection of any parasitic ova, oocysts, cysts, or larvae [15].

### 2.3. Blood Collection

Feline subjects were anesthetized using a combination of 12 mg/kg ketamine and 1 mg/kg xylazine intramuscularly [16]. Each ocelot was placed in lateral recumbency, and one forelimb was partially shaved to gain visual access to the cephalic vein. This area was swabbed with 70% alcohol, after which 5 ml of blood was removed via venipuncture using a 20-gauge hypodermic needle and syringe. Blood samples collected from all cats were placed in individually labeled red-topped tubes.

After blood collection, the ocelots were placed in recovery cages or areas prepared with bedding and low noise and light intensity to allow a smooth recovery without causing injury on awakening. The animals were monitored during recovery for any complications due to anesthesia.

Blood samples were transported in a cooler at 4°C to the haematology laboratory of the UWI-SVM. Sera were separated using centrifugation and stored at −80°C until further testing. Serum samples were tested by a latex agglutination test for the presence of antibodies against *Toxoplasma gondii* using the Toxotest-MT® (Eiken Chemical Co., Ltd., Tokyo, 110-8404, Japan) according to the manufacturer's instructions. The test is a modified indirect agglutination test predominantly utilized as a screening test with a sensitivity of 99% and specificity of 81% with confidence values of 94.4% and 92.5%, respectively. Based on the manufacturer's guidelines, a titer ≥64 was regarded positive.

## 3. Results and Discussion

### 3.1. Faecal Analysis

Of the 19 faecal samples tested, gastrointestinal parasites were observed in the faeces of 11 (57.9%) ocelots (Table 1). A total of eight parasitic genera were detected in gastrointestinal tracts of these felids by faecal flotation (Table 1). The most prevalent parasites were nematodes (ascarids and strongyles) followed by trematodes (*Schistosoma* spp.) and intestinal protozoa (*Balantidium* and *Isospora* spp.). A total of seven (36.8%) ocelots harboured two or more parasites (nematodes, trematodes, or protozoa), while three (15.8%) exhibited a mixed infection of both helminths and protozoa. Up to five parasite genera were observed in one felid. The mean number of parasite genera hosted by the infected ocelots in this study was 2.4.

**Figure 2:**
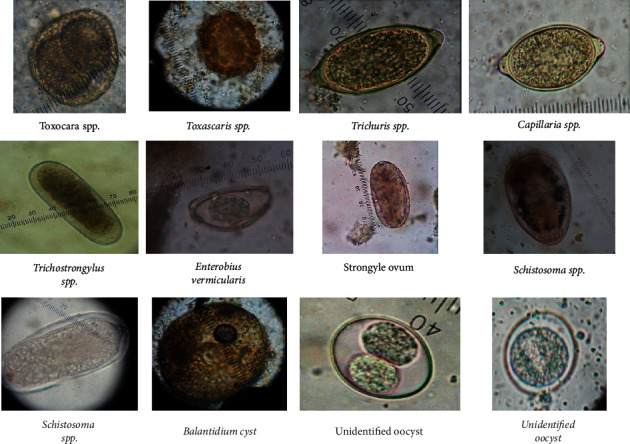
Images of gastrointestinal parasites identified among the captive ocelot population in Trinidad.

Previous reports from Trinidad detected one species of Acanthocephala (*Echinopardalis pardalis*), one cestode (*Diphyllobothrium* sp.), three nematode species (*Trichocephalus serratus*, *Molineus pardalis*, and trichostrongyle spp.), and an enteric arthropod (*Parrocephalus stilesi*) in noncaptive ocelots [13, 17–19]. However, in this current study, *Toxocara* spp., strongyle spp., *Trichostrongylus* spp., *Capillaria* spp., coccidian oocysts, *Balantidium* spp. cyst, and *Schistosoma* spp. have been identified in the faeces of infested ocelots (Figure 2). Since these cats are carnivorous and feed on a range of invertebrates, there is the possibility that some parasites were spurious [6]. It can also be suspected that the captive living conditions such as husbandry practices and prolonged confinement may also play a role in the rate of infestation of these parasites. Environmental contamination as a result of humans serving as fomited may also provide additional avenues for infestation particularly for those kept within a zoo environment where there are multiple visitors and zoo workers [20].

### 3.2. Serology

The sera of the thirteen [13] ocelots subjected to the latex agglutination test revealed three (23.1%) seropositive animals.

Seropositive animals were found in North, Central, and South Trinidad resulting in a total percent of positivity of 23.1%. This indicates that these animals have been exposed to *T. gondii* and are thus possible sources of infection to other intermediate hosts including humans. Based on location, the prevalence of *T. gondii* infection was approximately 7.69% with a higher presentation of adult females at 23.1% as opposed to males and immature animals at 0% in this sample population (Table 2).

The Fischer two-tailed exact test revealed a significant (*P* < 0.05) relationship between those from the North against the Central and South. There was no significant relationship with sex or age (adult vs. juvenile).

In recent times, a few of these animals have been utilized in the petting zoo and wildlife awareness programmes and interacted closely with the public. This could be a cause for concern, though minimal, for handlers and other persons in contact with infected ocelot faeces. An immunodeficient or pregnant animal may increase the risk of infection from the environment where actively infected ocelots defecate. The results of this survey indicate that these animals in particular should be regularly tested to determine the possible risk to their handlers and the wider population.

The small numbers tested were largely due to the small captive population which is a reflection of the small size of the island and local ocelot population. Some owners did not volunteer their animals for serological testing due to the risks associated with tranquilization. Therefore, statistical rigor was affected. However, it was found that finding a seropositive ocelot was higher for females (23.1%; *n* = 6) than for males (0%; *n* = 7).

The seroprevalence of *T. gondii* found in this study (23.1%; *n* = 13) was lower as compared to a national survey of captive ocelots in Brazil (57.7%; *n* = 168) [11]. This may be due to the small number of ocelots tested in this study. If this study is to be repeated using a larger number of captive ocelots, the data attained can be more reliable allowing more valid and direct comparisons to be made between both countries since Trinidad is geographically similar to the mainland of South America. A higher frequency of ocelots, 15/22 (68.18%), was found to be seropositive for *T. gondii* antibodies in a survey at a single location in Brazil [21]. In Mexico, two of three ocelots had antibodies to *T. gondii* at a zoo and 18 of 26 (69%) free ranging ocelots were found to be seropositive [22].

Although the sample size in this study was too small to detect any statistically significant differences within the ocelot population, the parasite diversity will aid in assessing the health status and possible parasite transmission by these cats. Another shortcoming of sample collection was the use of faeces that was not freshly voided. This sampling technique can potentially minimize parasite detection due to degradation of trophozoites or hatching of thin-shelled ova such as hookworms before analysis [23]. To detect these parasites effectively, it would be prudent to collect rectal faecal samples with analysis within 30–60 minutes of collection. This study thus serves as a pilot for future studies for the screening of parasites and other pathogens in the wild ocelot population in Trinidad and Tobago.

## 4. Conclusions

More than 50% of the captive ocelot population in Trinidad harbour endoparasites and approximately 25% are seropositive for *T. gondii*. Care must be taken when handling the faeces of captive ocelots, and proper waste disposal must be implemented to prevent environmental contamination and infection of other animals and humans with these parasites.

## Figures and Tables

**Table 1:** Prevalence of gastrointestinal parasites among the captive ocelot population in Trinidad.

Parasites detected	Number positive (*n* = 19)	Prevalence (%)
Nematodes		
Ascarids	7	36.8
Strongyles	6	31.6
*Trichostrongylus* spp.	2	10.5
*Capillaria* spp.	3	15.8
*Trichuris* spp.	2	10.5

Trematodes		
*Schistosoma* spp.	3	15.8

Protozoans		
*Balantidium coli*	1	5.3
Unidentified oocyst	2	10.5

**Table 2:** Serological results for *Toxoplasma gondii* in 13 individual ocelots based on the percent of positivity regarding the gender, geographical location in Trinidad, adult, and immature ocelots.

	Male (*n* = 7)	Female (*n* = 6)	North (*n* = 8)	Central (*n* = 3)	South (*n* = 2)	Adult (*n* = 11)	Immature (*n* = 2)
Positive (%)	0	23.1	7.69	7.69	7.69	23.1	0
Negative (%)	53.8	23.1	53.8	15.4	7.69	61.5	15.4

## Data Availability

The parasitology and serology results data used to support the findings of this study are included within the article.
